# Abstractive Arabic Text Summarization Based on Deep Learning

**DOI:** 10.1155/2022/1566890

**Published:** 2022-01-11

**Authors:** Y. M. Wazery, Marwa E. Saleh, Abdullah Alharbi, Abdelmgeid A. Ali

**Affiliations:** ^1^Faculty of Computers and Information, Minia University, Minia, Egypt; ^2^Department of Information Technology, College of Computers and Information Technology, Taif University, P. O. Box 11099, Taif 21944, Saudi Arabia

## Abstract

Text summarization (TS) is considered one of the most difficult tasks in natural language processing (NLP). It is one of the most important challenges that stand against the modern computer system's capabilities with all its new improvement. Many papers and research studies address this task in literature but are being carried out in extractive summarization, and few of them are being carried out in abstractive summarization, especially in the Arabic language due to its complexity. In this paper, an abstractive Arabic text summarization system is proposed, based on a sequence-to-sequence model. This model works through two components, encoder and decoder. Our aim is to develop the sequence-to-sequence model using several deep artificial neural networks to investigate which of them achieves the best performance. Different layers of Gated Recurrent Units (GRU), Long Short-Term Memory (LSTM), and Bidirectional Long Short-Term Memory (BiLSTM) have been used to develop the encoder and the decoder. In addition, the global attention mechanism has been used because it provides better results than the local attention mechanism. Furthermore, AraBERT preprocess has been applied in the data preprocessing stage that helps the model to understand the Arabic words and achieves state-of-the-art results. Moreover, a comparison between the skip-gram and the continuous bag of words (CBOW) word2Vec word embedding models has been made. We have built these models using the Keras library and run-on Google Colab Jupiter notebook to run seamlessly. Finally, the proposed system is evaluated through ROUGE-1, ROUGE-2, ROUGE-L, and BLEU evaluation metrics. The experimental results show that three layers of BiLSTM hidden states at the encoder achieve the best performance. In addition, our proposed system outperforms the other latest research studies. Also, the results show that abstractive summarization models that use the skip-gram word2Vec model outperform the models that use the CBOW word2Vec model.

## 1. Introduction

Through the past two decades, there is a rapid and wide increase in the amount of data available on the Internet such as news, articles, journals, book reviews, etc. So, automatic text summarizing systems are extremely needed to extract important information from the enormous amount of available text instead of reading the whole text [1]. In general, text summarization can be defined as the process of generating a short text from a longer text document by using software, where this short text is a summary of the major parts of the original document [2]. Text summarization can be classified, based on the three angles of observation. The first angle is based on the input type, where the summarization process can be categorized into a single-document summarization or multidocument summarization. In the single-document summarization, the input is only one document, and the summary is generated from this document while in multidocument summarization, the input is multiple documents, and the summary should contain information from all of these documents. The second angle is based on the context, where the summarization process can be categorized into generic, query-driven, or domain-specific summaries. Generic summaries use only the original document(s) while query-driven summaries focus on returning the important information related to a query from the user or that answers a user's query. Domain-specific summaries use some domain knowledge to make a summary [3]. The last and the most important angle of text summarization is based on the output type, where there are two types, extractive and abstractive summarization. In extractive summarization, the summary is created from sentences or phrases in the source document(s) based on statistics and linguistic features, while abstractive summarization expresses the ideas in the source documents using different words based on the real semantics of the text [1]; [3]. Furthermore, abstractive summarization is more complicated than extractive summarization because abstractive summarization requires semantic analysis of the text that can be achieved by using machine learning techniques and advanced natural language processing (NLP) [4]. However, abstractive summarization is better, since it is like a summary that is written by humans, so it is more meaningful [5].

**Figure 1:**
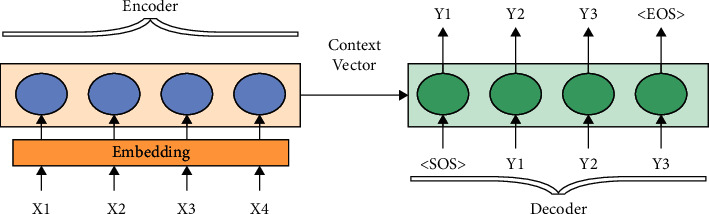
Sequence-to-sequence model [11].

Recently, deep learning methods provided significant improvements in important tasks like text translation [6]; sentiment analysis [7]; and text summarization and others fields [8]. Also, the important feature of using deep neural networks is it takes advantage of big datasets to improve their results [9]. The new text summarization methods are based on a sequence-to-sequence framework of the encoder-decoder model. This model consists of two parts, encoder and decoder. The encoder reads a new token from the input sequence at each time step and updates the hidden states depending on this token. After reaching the last token of the input sequence, the encoder produces the context vector with a fixed length as the representation of the input, regardless of the input length. The context vector is the final hidden state that is used to initialize the decoder. The decoder, the second component of sequence-to-sequence model, is initialized with hidden states (context vector) from the encoder as a first hidden state, as well as 〈SOS〉 token as the start point of the output sequence. However, the decoder is trained to produce a new sequence with a fixed length. At each time unit produces a new word from the vocabulary by giving the previously generated word [10]; [9] as shown in Figure 1, where the last hidden state of the encoder is fed as input to the decoder with the start token 〈SOS〉 [11]. This model has been used in several NLP applications, such as text summarization and machine translation. In text summarization, the document that needs to be summarized is the input sequence, and the summary is the output while, in machine translation, the sentence in a specific language is the input sequence, and the corresponding sentence in another language is the output [12]; [11].

**Figure 2:**
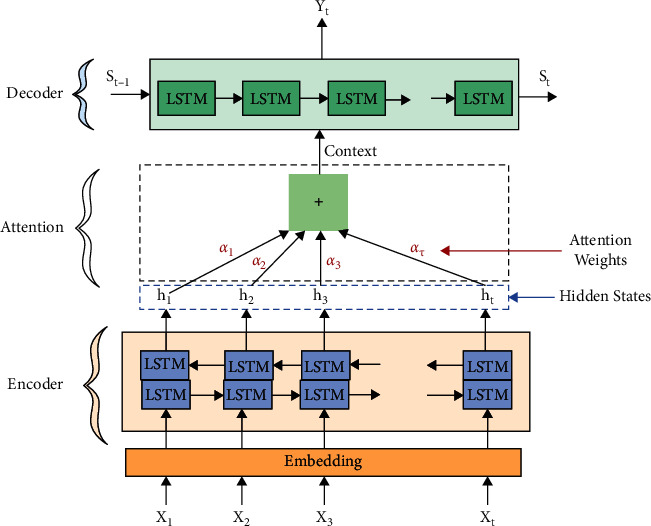
Sequence-to-sequence with attention [12].

The main drawback of this model is it encodes the entire input sequence into just one small vector (context vector), so it is difficult to summarize a long sequence. To solve this problem, an attention mechanism was created by Dzmitry Bahdanau et al. [12]. The main idea of attention is it only pays attention to some words of input that are most relevant information instead of the entire sequence as shown in Figure 2, where the hidden states of the encoder are fed as input to attention and context from attention is fed to the decoder at each output time step [12].

There are two different types of attention mechanisms such as global and local attention. The difference between them depends on the derivation way of the context vector. In global attention, the derivation of the attended context vector depends on all the hidden states of the encoder while it depends on only a few hidden states of the encoder in local attention [13].

Most of the existing work in this area focuses on the English language but it still lacks with the Arabic language due to its complexity, including the Arabic diglossia, the large variety in dialects, and the complex morphology of the Arabic language [1, 2], [1]. Moreover, a lot of existing solutions in this area are being carried out in extractive summarization and few of them are being carried out in abstractive summarization especially in the Arabic language [1, 2]; [1]. On the other hand, Arabic is the national language for 22 countries and more than 300 million people speak Arabic (Al-Saleh and Menai [2]. Therefore, Arabic summarization systems are highly needed these days.

Therefore, the first contribution in this research is to propose an abstractive Arabic text summarization system that is based on deep learning. In particular, the proposed system is based on a sequence-to-sequence model with a global attention mechanism to generate an abstractive summary for Arabic news. In addition, AraBERT preprocess [14] has been applied in the data preprocessing stage that helps the model to understand the Arabic words and achieves state-of-the-art results. Also, early stopping has been applied to stop training the model at the right time. In the second contribution, several deep artificial neural networks have been used for developing the proposed system to investigate which of them achieved the best performance, namely, GRU, LSTM, and BiLSTM. In the third contribution, we prove that the generated summary's quality is affected highly by the word embedding's quality by applying the skip-gram and the continuous bag of words (CBOW) word2Vec word embedding models and comparing between them. The rest of the paper is organized as follows: Section 2 presents the related work, while in Section 3, the proposed system is explained in detail. In Section 4, the experimental results and evaluation are discussed. Finally, in Section 5 the conclusion and future work are covered.

## 2. Related Work

Several studies were accomplished in the literature for Arabic text summarization, but most of them were extractive summarization covering single- and multidocuments. These studies have been based on specifying the important parts of the text according to three approaches, which are symbolic, numerical, and hybrid approach [1]; [2]. On the other hand, there is not much work done until now in abstractive summarization in other languages particularly Arabic language [11]. Therefore, we will start with the recent works for Arabic abstractive summarization and then move to English abstractive summarization.

In general, there are two approaches for abstractive summarization which are semantic-based and structured approaches. The first approach focuses on identifying noun and verb phrases by processing linguistic data to summarize the text. Methods that use a semantic-based approach involve the information item method, multimodal semantic method, and semantic graph-based methods. However, in the second approach, the important information of documents is encoded by using lead and body phrases, ontology, including tree, and template and rule-based schemas [14].

In Arabic text summarization, there is only one study in the literature that uses a rich semantic graph (RSG) for a single Arabic text document abstractive summarization [15]. The RSG [16] is an ontology-based representation that represents nouns and verbs of the input document as graph nodes, while edges represent topological and semantic relations between the nodes. The system consists of three phases: first, an RSG graph is created for the source document, and then the generated RSG graph is reduced to a more abstracted graph. At last, the abstractive summary is generated from the abstracted graph. A major drawback of this system is it is based on a manually built ontology that is a time-consuming task [15]. Another research for abstractive summarization in the Arabic language appeared in 2018 [4] that had four phases to summarize. First, the input document is broken into segments that are topically coherent multiparagraph subparts. Then, headline keywords are generated for each segment. After that, a primary extractive summary is generated by extractive summarizing for each segment. Finally, the sentence reduction technique is applied to generate the abstractive summary. The drawback of this system is it depended on extractive summarization, so it is not a purely abstractive method.

Lately, deep learning has provided excellent results and it has been extensively employed in recent years in important tasks such as text translation and sentiment analysis. Deep learning was utilized in Arabic abstractive text summarization for the first time in 2017 by Khoja et al. [17], where two models were introduced. The first model uses a standard sequence-to-sequence architecture, and the second model uses a sequence-to-sequence model with attention. However, the used dataset is relatively small.

In 2019, a study on abstractive text summarization for multiple languages including the Arabic language appeared [18]. In this study, multiple models were applied in multiple datasets for English and Arabic and then compared between them. These models are simple sequence-to-sequence with attention, Pointer-Generator, Scheduled-Sampling, and Policy-Gradient. In addition, a novel advanced cleaning technique was introduced that increased the relevancy of the vocabulary and also the efficiency of the text summarization. This technique was applied to the Arabic dataset.

In 2020, two studies appeared in Arabic abstractive text summarization, the first one was by Dima Suleiman and Arafat Awajan [13], in which they introduced a model that consists of two layers at the encoder which are the input text layer and the name entities layer, while one layer at the decoder. Both the encoder and the decoder use LSTM, but bidirectional LSTM is used in the encoder while unidirectional LSTM is used in the decoder. They used one of the AraVec pretrained word embedding models for the embedding layer. The experiments were conducted in a dataset that was collected and preprocessed to be suitable for abstractive summarization. For evaluation, ROUGE1 and ROUGE1-NOORDER were used as evaluation measures where their values were 38.4 and 46.4, respectively. However, the collected dataset is small and not available to the public for allowing other studies to compare with them. The second one was done by Molham Al-Maleh and Said Desouki Al-Maleh and Desouki [9], in which a new dataset for the Arabic language was built, and then the abstractive sequence-to-sequence with the attention mechanism (baseline) was applied on TensorFlow. After that, the copy mechanism was added to match the pointer-generator model and take advantage of both abstractive and extractive approaches that improved their results. At last, coverage and length penalties were applied on both models. ROUGE F1 was used as an evaluation measure with a value of 44.23.

Moving on to abstractive summarization in the English language, deep learning was utilized in English abstractive text summarization for the first time in 2015 by Rush et al. [19], in which three types of encoder were proposed including a bag of words, convolution, and attention-based encoders. In addition, the local attention mechanism was used by the decoder which conditions every word of the summary to the input sentence. Furthermore, beam search was used to select the best *k* target words. Gigaword dataset was used for training while DUC-2003 and DUC-2004 datasets were used for testing. There were several preprocessing stages performed on the datasets such as using lower case letters, UNK token to represent the least frequently words, tokenization, and using the symbol to replace all digits. To evaluate the quality of the generated summary, ROUGE1, ROUGE2, and ROUGE-L were used where the value of ROUGE1 was 28.18 while the values of ROUGE2 and ROUGE-L were 8.49 and 23.81, respectively.

Chopra et al. [20] introduced RAS (Recurrent Attentive Summarizer) that is an extension of abstractive sentence summarization [19] by using a Recurrent Neural Network (RNN) architecture instead of using a feed-forward neural network. Gigaword dataset was used for training while the DUC-2004 dataset was used for evaluation. ROUGE1, ROUGE2, and ROUGE-L were used to evaluate the generated summary quality, and the results were 28.97, 8.26, and 24.06, respectively.

Nallapati et al. [21] proposed an abstractive text summarization model that uses an attention mechanism with RNN encoder-decoder architecture. The encoder consists of two layers bidirectional GRU-RNN while the decoder layer consists of one layer unidirectional GRU-RNN. The first layer in the encoder represents the word level while the second layer is used for the sentence level. Moreover, the softmax layer in the decoder is used to generate the summary words. The word embedding of the words and several features of input text including the name entities, part of speech tagging, and TF-IDF were fed to the encoder. Word2vec was used to convert the words into vectors. For training the model, DUC, Gigaword, and CNN/Daily datasets were used while ROUGE-1, ROUGE-2, and ROUGE-L were used for evaluating the quality of generated summaries where their values were 35.46, 13.3, and 32.65, respectively.

Zhou et al. [22] proposed a Selective Encoding for Abstractive Sentence Summarization (SEASS) model that uses a selective encoding model to extend the sequence-to-sequence framework for abstractive sentence summarization. It consists of an encoder, decoder, and selective gate. SEASS model consists of a bidirectional GRU encoder and a unidirectional GRU decoder. The selective gate generated the representation of the sentences' words. DUC 2004, Gigaword, and MSR-ATC datasets were used for training and testing. Furthermore, the beam search is used to select the best target word. Finally, ROUGE1, ROUGE2, and ROUGE-L were used to evaluate the quality of generated summaries and their values were 36.15, 17.54, and 33.63, respectively.

The dual-attention sequence-to-sequence framework was proposed by Cao et al. [23]. Their model consists of two encoders with bidirectional GRU and one decoder that has a gate network of dual attention. Furthermore, two context vectors are generated and merged by the decoder instead of one context vector. Gigaword dataset was used for experimentation showing that their model greatly reduces fake summaries by 80%. To evaluate the quality of the generated summary, ROUGE1, ROUGE2, and ROUGE-L were used, and their values were 37.27, 17.65, and 34.24, respectively.

A hybrid pointer-generator architecture with coverage was proposed by See et al. [24] that uses a hybrid pointer-generator network to copy words from the source text via pointing and uses coverage to keep track of what is summarized to prevent repetition. Their model was applied to the CNN/Daily Mail, and ROUGE1, ROUGE2, and ROUGE-L were used to evaluate the quality of the generated summary with values 39.53, 17.28, and 36.38, respectively.

As we can see there are only four research studies in abstractive Arabic text summarization, this motivated us to focus on applying abstractive text summarization to the Arabic language and attempting to enhance the Arabic text summarization quality. In the next section, the proposed system is explained in detail.

## 3. Proposed System

**Figure 3:**
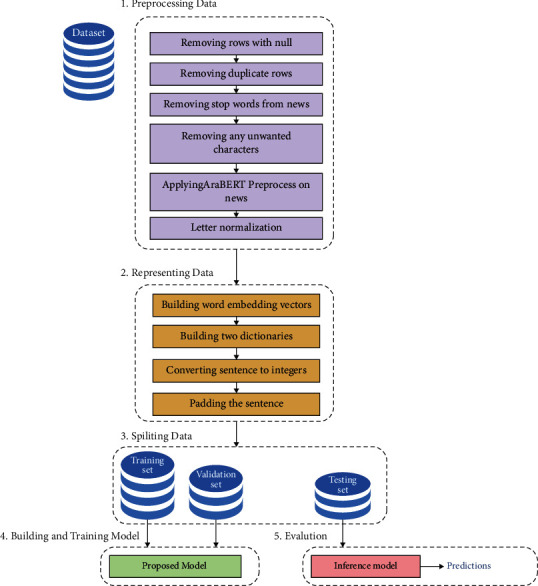
The architecture of the proposed Arabic text summarization system.

The different components and steps that have been used by our system are described in this section. The proposed system consists of five stages which are as follows: preprocessing data, representing data, splitting data, building and training model, and evaluation as shown in Figure 3. The five stages of the proposed system are described in detail as follows.

### 3.1. Preprocessing Data

This step aims to clean data and convert it into a coherent form to easily handle it. First, rows with NULL values in either new content or summary have been removed, and duplicate rows also have been removed. Then, we have applied the following steps:

1. Removing stop words only from the news content such as ' 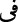 ' 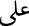
' ' 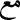 ', and etc. to less the data and train the model faster because they are not very relevant in training the model. However, stop words have remained in the summaries because they are important for the model to make predicted summaries more like natural phrases.
2. Removing any unwanted characters such as punctuation, URLs, slash, etc.
3. Applying AraBERT preprocess [25] to remove Arabic additions from words such as ' 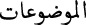 ' converts to ' 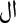 ' ' 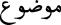 ' , and ' 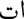 '. We remove these additions to reduce number of words.
4. Applying letter normalization to unify the letters that appear in different forms such as replacing 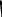 in {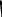

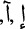
} with {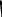}, {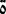} with {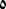}, and {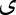} with {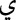}.

### 3.2. Representing Data

Due to deep learning and neural networks just accept numbers as input but a text is a string (not a number), word embedding is used to solve this problem. In our implementation, word embeddings have been created using the word2vec [26]; and the skip-gram architecture has been used with Windows 10. A dataset of unique Arabic news has been used to train and build the word embeddings by concatenating each new content with its summary. The dimension size of the building word embedding vectors that represent each word has been 150. After that, two dictionaries have been built to convert words to integers that represent their indexes for input and output sequences. To reduce the size of vocabulary, we have used only words that have been in word embeddings or that have appeared more than or equal to 10 times to build a dictionary. This reduces computation time and complexity. In addition, special tokens have been added to the dictionary such as 〈UNK〉, 〈PAD〉, 〈EOS〉, and 〈SOS〉 where 〈UNK〉 token has been used to replace the less frequent words or unknown words, 〈PAD〉 token has been used to make padding for the short sentences, 〈SOS〉 token has been used as start token of a sentence that fed into the decoder, and 〈EOS〉 token has been used as end token of a sentence.

To help train the model faster, the length of texts and summaries has been inspected to fix the maximum length of the news contents and summaries by taking the majority of news contents' length and summaries' length. This reduces much extra padding and computation. Moreover, some news does not have been included, if there would have been more than 1 UNK in the news content or any UNKs in the summary. This has been done to ensure that the model has been built with meaningful data. Finally, each sentence in news contents and summaries has been converted to a set of integers by using two dictionaries and padded to match the longest sentence in the training set.

### 3.3. Splitting Data

In this step, the dataset has been splatted into three sets, the training set, validating set, and testing set. The training set has been used for training our model, the validation set has been used for validating the model, and the testing set (unseen set) for testing and evaluating our model.

### 3.4. Building and Training Model

**Figure 4:**
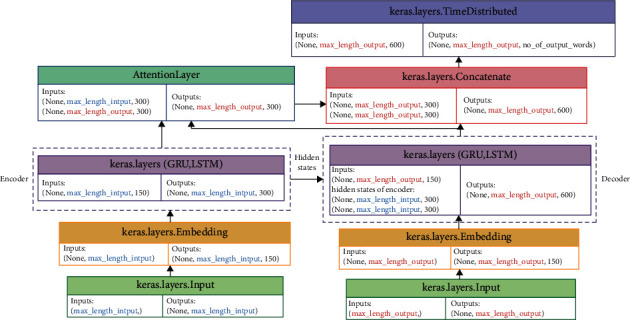
The sequence-to-sequence model with LSTM and GRU layer at the encoder and the decoder.

**Figure 5:**
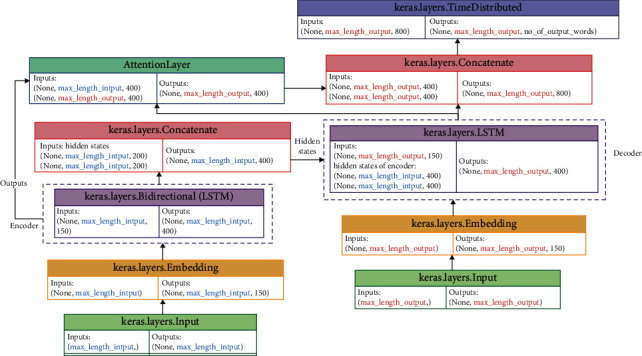
The sequence-to-sequence model with BiLSTM layer at the encoder and LSTM at the decoder.

The sequence-to-sequence framework of the encoder-decoder with attention mechanism is used to generate an abstractive summary, based on the news content or article content as the original text. Our aim was of developing the sequence-to-sequence model using several deep artificial neural networks to investigate which of them achieves the best performance. Deep artificial neural networks, including, Gated Recurrent Units (GRU), Long Short-Term Memory (LSTM), and Bidirectional Short-Term Memory (BiLSTM) are used to building the proposed model. The building model stage consists of building three components which are the encoder, the decoder, and global attention. A global attention mechanism is used instead of local since it provided better results. Unfortunately, Keras does not formally support the attention layer, so a third-party implementation is used. Also, we are used a different number of hidden states layers at the encoder while one hidden states layer at the decoder to investigate the effect of the number of layers on the generated summary's quality. The architecture of the proposed model with the shape of input and output for each layer is shown in Figure 4 for LSTM and GRU while Figure 5 for BiLSTM.

In Figures 4 and 5, the input for the input layer is the input sequence with a max length at the encoder side while it is the target sequence with a max length at the decoder side. Then, the output of the input layer is fed into the embedding layer that produces the word embeddings for the input sequence at the encoder side while it produces the word embeddings for the target sequence at the decoder side. The output of the embedding layer is fed into the encoder that has some GRU, LSTM, or BiLSTM hidden states layers. The encoder produces two things in the case of GRU which are the output and hidden state while it produces three things in the case of LSTM which are the output, hidden state, and cell state. On the other hand, the encoder in the case of BiLSTM produces five things including the output, two hidden states for the forward and backward sequence, and two cell states for the forward and backward sequence. After that, the hidden state is fed into the decoder in the case of GRU while the hidden state and cell state are fed into the decoder in the case of LSTM. Otherwise, in the case of BiLSTM, the two last hidden states are concatenated together using the concatenate layer and the two last cell states also; then they are fed into the decoder, so the input of the decoder is three vectors, one from the embedding layer and two from the encoder as shown in Figure 5. Then, the output from the encoder and the decoder is fed into the attention layer that is used to focus on the input parts that has a highly significant effect on the output and produced the context vector. The proposed model has used a global attention mechanism since it provided better results than a local attention mechanism [13]. Finally, the context vector from the attention layer and the output from the decoder are concatenated together and fed into the TimeDistributed layer that is a softmax-activated dense layer to receive the vocabulary distribution, attaching probabilities to each word in the vocabulary. The word with the highest probability is then chosen as the next output. All variations of the proposed model have been trained by using a training set (which is 90% of the dataset) and validated using a validation set (which is 9% of the dataset) through 50 epochs and a batch size of 40. Sparse categorical cross-entropy has been used as a loss function because it overcomes any memory issues. Furthermore, early stopping has been used for stopping training the neural network at the right time by monitoring val_loss, so our models have been stopped training once the validation loss has increased after two iterations.

### 3.5. Evaluation

For testing and evaluating all variations of the proposed model, the testing set is fed into the inference model as shown in Figure 3, in which the decoder is a little different than in the training. This difference is only in the input for the decoder hidden states where the inputs at time *t* are the output of the previous hidden state and the word embedding of the next word in reference summary (target summary) during the training. While during testing, there is no reference summary, so the inputs for the hidden state are the output of the previous hidden state and the word embedding of the previously generated word of the predicted summary. Furthermore, the input for the first hidden state in both training and testing is the word embedding of 〈SOS〉 token and the output hidden states from the encoder.

### 3.6. Evaluation Metrics

For evaluating the quality of all variations of the proposed model, two standard metrics are used including ROUGE and BLUE which are nondifferentiable metrics qualified for comparing the generated summary to the reference summary. ROUGE (Recall-Oriented Understudy for Gisting Evaluation) is a set of metrics and a software package used for evaluating machine translation and automatic text summarization and it is a recall-based evaluation metric [27]. ROUGE measures the number of words from the reference summary that appeared in the generated summary using n-gram overlap between them. We compute precision, recall, and f1-measure scores for ROUGE-1, ROUGE-2, and ROUGE-L, where ROUGE-1 measures the word-overlap, ROUGE-2 measures bigram-overlap, and ROUGE-L measures the longest common sequence between the reference summary and the generated summary.

F1-measure provides the harmonic mean between precision and recall and it is computed as

(1) $$\begin{array}{c} F1-\text{measure}=2\cdot \frac{\text{precision}\times \text{recall}}{\text{precision}+\text{recall}}, \end{array}$$

where precision measures the percentage of n-grams from the generated summary that is relevant to the reference summary and it is computed as

(2) $$\begin{array}{c} \text{precision}=\frac{n{\text{gram}}_{\text{Ref}}\cap n{\text{gram}}_{\text{Gen}}}{n{\text{gram}}_{\text{Gen}}}, \end{array}$$

while recall shows how far the generated summary fulfills the reference summary and it is computed as

(3) $$\begin{array}{c} \text{recall}=\frac{n{\text{gram}}_{\text{Ref}}\cap n{\text{gram}}_{\text{Gen}}}{n{\text{gram}}_{\text{Ref}}}, \end{array}$$

where n-gram Ref is the number of n-grams in reference summary and n-gram Gen is the number of n-grams in generated summary. On the other hand, F-measure for ROUGE-L is computed as follows [27]:

(4) $$\begin{array}{c} {\text{F}-\text{measure}}_{\text{LCS}}=\frac{\left(1+\beta^{2}\right){\text{recall}}_{\text{LCS}}{\text{precision}}_{\text{LCS}}}{{\text{recall}}_{\text{LCS}}+\beta^{2}{\text{precision}}_{\text{LCS}}},\\ {\text{precision}}_{\text{LCS}}=\frac{\text{LCS}\left(X,Y\right)}{m},\\ {\text{recall}}_{\text{LCS}}=\frac{\text{LCS}\left(X,Y\right)}{n}, \end{array}$$

where LCS (*X*, *Y*) is the length of a longest common subsequence of *X* and *Y*, *m* and *n* are the length of *X* and *Y*, while ß is precision_Lcs_/recall_LCs_.

BLEU (Bilingual Evaluation Understudy) compares generated summary to one or more reference summary by computing the number of words from generated summary that appeared in the reference summary [28]. BLEU is a precision-based evaluation metric, and its score is computed as follows [29].

First, compute a brevity penalty which looks for the reference with the most similar length by

(5) $$\begin{array}{c} \text{BP}=\left\{\begin{array}{cc} 1, & \text{if }c>r,\\ e^{1-r/c}, & \text{if }c\leq r, \end{array}\right) \end{array}$$

where *c* and *r* are a candidate summary and a reference summary, respectively. Finally, the BLEU score is computed by

(6) $$\begin{array}{c} \text{BLEU}=\text{BP}*\;\exp\left(\overset{N}{\underset{n=1}{\sum }}w_{n}\log\;p_{n}\right), \end{array}$$

where *p*_*n*_ is the n-gram precisions score and *w*_*n*_ is positive weights [29].

## 4. Experiments

### 4.1. Experiment Setup

The experiments have been implemented using *Python* with Keras and run-on Google Colab Jupiter notebook with a Tesla P100-PCIE-16 GB GPU and 27.4 GB RAM. We have used a “Keras” library due to it focuses on being modular, user-friendly, and extensible. In addition, a Keras is a high-level neural network library that runs on top of TensorFlow [30].

### 4.2. Datasets

We have dealt with two datasets which are as follows:

1. The Arabic Headline Summary (AHS) dataset 1
2. The Arabic Mogalad_Ndeef (AMN) dataset 2

The AHS dataset was used in Al-Maleh and Desouki [9] that contains approximately 300*k* Arabic articles and their titles. We consider the news content as the original text and its titles as a summary for it. While the AMN dataset was used in Zaki et al. [18] that contains approximately 265*k* Arabic news, we focus on two fields from the dataset, which are the news content and its summary.

### 4.3. Results

In this section, the experimental results show the effect of the deep artificial neural networks such as the GRU, LSTM, and BiLSTM, and its number of layers, the AraBERT preprocess, and the quality of word embedding model on the generated summary's quality. We have applied our experiments to the AHS and AMN datasets. The testing set has been used for evaluation and all results are the average. Furthermore, a comparison with other research studies is conducted to confirm the effectiveness of our proposed system.

After applying the preprocessing data stage on the AHS dataset and AMN dataset, they become 294 835 and 254 107 unique Arabic articles and news with their summaries, respectively. Then, representing data stage has been applied, so the total number of unique words and used words and their percentage are shown in Table 1.

Due to limitations in computing resources, we have removed news that has news content length more than 412 and 42 in the summary length for the AHS dataset while 1786 and 112 for the AMN dataset. In addition, we have removed news that has more than 1 UNK in the news content or any UNKs in the summary, so the datasets become 247 663 and 196 874 in AHS dataset and AMN dataset, respectively. Then the dataset is splatted into three sets as shown in Table 2.

Tables 3 and 4 show the results of applying GRU, LSTM, and BiLSTM for the proposed model with one, two, and three hidden layers at the encoder to the AHS and AMN datasets. It can be observed that using two layers of GRU and LSTM at the encoder achieves the highest average values of F-measure for ROUGE-1, ROUGE-2, and ROUGE-L in addition to BLEU. Therefore, applying GRU and LSTM with two hidden layers at the encoder is the best for the sequence-to-sequence model among other GRU and LSTM using one and three hidden layers to achieve the best performance. On the other hand, it can be observed that using three layers of the BiLSTM at the encoder achieves the highest average values of F-measure for ROUGE-1, ROUGE-2, and ROUGE-L in addition to BLEU. Therefore, applying BiLSTM with three hidden layers is the best for the sequence-to-sequence model among other BiLSTM models where it achieved the best performance.

Furthermore, for studying the effect of AraBERT preprocess on the generated summary's quality, we implement the proposed system with AraBERT preprocess and without AraBERT preprocess on AHS and AMN datasets and compare the results as shown in Table 5. It can be observed that the AraBERT preprocess provides better results than without using it. As a result, the AraBERT preprocess plays an important role in the preprocessing data stage.

Moreover, a comparison between the skip-gram and the continuous bag of words (CBOW) word2Vec word embedding models has been conducted as shown in Table 6. The results show that abstractive summarization models that use the skip-gram word2Vec model outperform the models that use the CBOW word2Vec model. As a result, the generated summary's quality is affected highly by the word embedding's quality.

For comparison with other Arabic research studies that uses deep learning for text summarization, we compare our proposed system by applying three layers of BiLSTM at the encoder with two latest Arabic research studies based on deep learning (Dima Suleiman and Arafat Awajan [11]; ENCODER [13]; and Al-Maleh et al. Al-Maleh and Desouki [9], as shown in Table 7.

According to the comparison results in Table 7, it is found that our proposed system has the best F1-measure value comparing with other research studies [9] which means that the summary's quality of our proposed system is the highest.

### 4.4. Discussion

**Figure 6:**
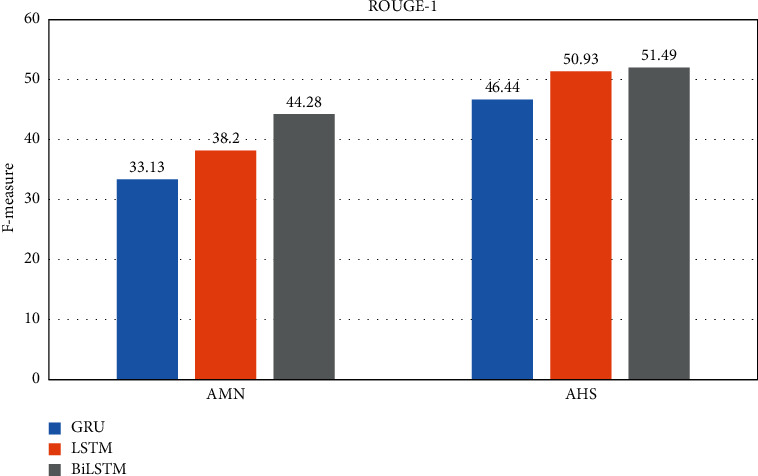
The best testing performance by ROUGE-1.

**Figure 7:**
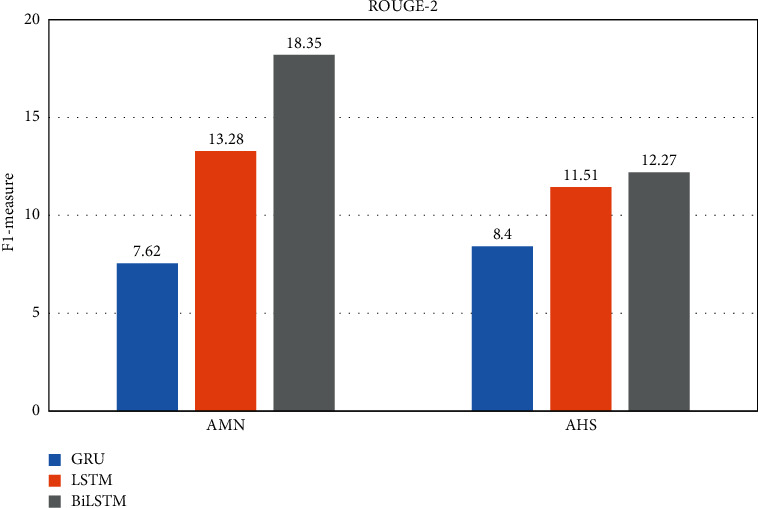
The best testing performance by ROUGE-2.

**Figure 8:**
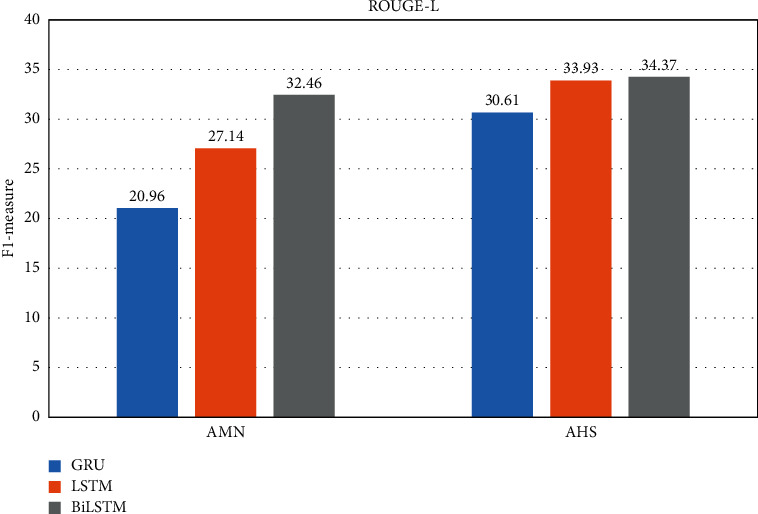
The best testing performance by ROUGE-L.

**Figure 9:**
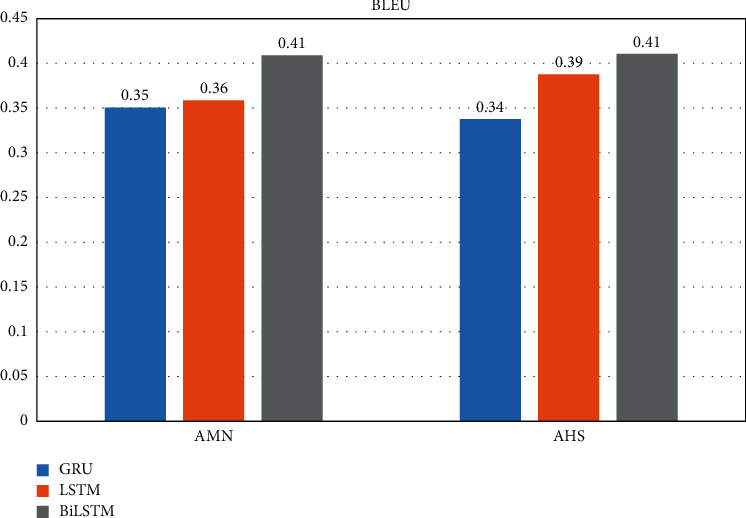
The best testing performance by BLEU.

By considering the experimental results seen in Tables 3 and 4, the best performance of the GRU and LSTM is the using two hidden layers at the encoder. For BiLSTM, three hidden layers have achieved the best performances. It is observable from Figures 6–9 that the proposed system with applying the BiLSTM outperforms the GRU and LSTM. Thus, we can conclude that three layers of the BiLSTM hidden states at the encoder achieve the best performance.

Also, from our experimental tests, we have found that the applying of AraBERT preprocess in preprocessing data stage has played an essential role in achieving the best performance as shown in Table 5.

Furthermore, from our experimental tests, we have found that the building of word embeddings using the skip-gram word2vec is better than the continuous bag of words (CBOW) word2vec as shown in Table 6. As a result, the generated summary's quality is affected highly by the word embedding's quality.

Finally, there are three examples of a generated summary using the proposed system by applying BiLSTM with three hidden layers at the encoder to prove the generated summary's quality. The first and second examples are randomly drawn from the testing set of the AHS dataset while the third example is from the testing set of the AMN dataset. The articles or news content, the reference summary, and the generated summary by using the proposed system are shown in Table 8.

## 5. Conclusion

In this paper, an abstractive Arabic text summarization system based on sequence-to-sequence has been proposed. The core merit of this paper is investigating in three directions that impact the generated summary's quality. The first direction is the type of deep artificial neural network and its number of layers that are used to implement the encoder and the decoder. We found that that three layers of BiLSTM hidden states at the encoder achieve the best performance. The second direction is the way of preprocessing data and we found that the AraBERT preprocess has played an essential role in achieving the best performance. The third direction is the word embedding model that is used and the results showed that the skip-gram word2vec generated better summary quality than the CBOW word2vec model.

We are looking forward to applying reinforcement learning algorithms and combining reinforcement learning techniques with deep learning models to improve the quality of the generated summary.

## Figures and Tables

**Table 1:** Total number of unique words, used words, and their percentage for input and output sequences.

Dataset	Dictionary	Unique words	Used words	Percentage
AHS	Input	87507	47464	54.24
	Output	41859	28012	66.92

AMN	Input	186191	10 626	56.73
	Output	106935	72882	68.16

**Table 2:** The three sets from the AHS and AMN datasets with number of tokens for input and output sequence.

dataset	Training set	Validation set	Testing set	No. of tokens	
				Input	Output
AHS	222,896	22,290	2,477	92	13
AMN	177,186	17,719	1,950	372	26

**Table 3:** The result of applying GRU, LSTM, and BiLSTM with different layers at the encoder to the AHS dataset.

Model	No. of layers	ROUGE-1			ROUGE-2			ROUGE-L			BLEU
		Precision	Recall	F1	Precision	Recall	F1	Precision	Recall	F1	
GRU	1	49.44	44.12	45.17	8.0	7.13	7.35	34.05	31.31	29.81	0.32
	2	**50.35**	**45.65**	**46.44**	**9.1**	**8.16**	**8.4**	**34.52**	**32.2**	**30.61**	**0.34**
	3	49.87	44.0	45.37	8.08	7.24	7.47	34.53	31.4	30.14	0.32

LSTM	1	53.76	48.87	50.1	11.78	10.73	10.99	37.1	34.1	33.44	0.38
	2	**54.82**	**49.53**	**50.93**	**12.27**	**11.29**	**11.51**	**37.71**	**34.46**	**33.93**	**0.39**
	3	53.04	47.99	49.3	11.47	10.29	10.64	37.15	33.78	33.26	0.37

BiLSTM	1	54.16	49.84	50.8	12.65	11.63	11.89	37.51	34.87	34.05	0.39
	2	55.12	50.2	51.46	13.13	11.92	12.25	38.0	35.04	34.44	0.40
	3	**54.95**	**50.48**	**51.49**	**13.1**	**12.01**	**12.27**	**37.84**	**35.19**	**34.37**	**0.41**

The highest result is given in bold.

**Table 4:** The result of applying GRU, LSTM, and BiLSTM with different layers at the encoder to the AMN dataset.

Model	No. of layers	ROUGE-1			ROUGE-2			ROUGE-L			BLEU
		Precision	Recall	F1	Precision	Recall	F1	Precision	Recall	F1
GRU	1	19.89	17.81	18.17	1.28	1.14	1.16	13.62	12.88	12.11	0.22
	2	**39.04**	**30.77**	**33.13**	**8.66**	**7.15**	**7.62**	**25.05**	**21.61**	**20.96**	**0.35**
	3	35.31	26.81	29.23	6.33	5.2	5.54	22.14	18.57	18.1	0.32

LSTM	1	39.41	32.66	34.78	11.47	9.91	10.38	26.76	23.52	23.28	0.34
	2	**43.47**	**36.23**	**38.2**	**14.5**	**12.71**	**13.28**	**31.13**	**27.01**	**27.14**	**0.36**
	3	36.26	32.35	33.34	10.36	9.61	9.76	25.68	23.66	23.05	0.349

BiLSTM	1	48.41	41.38	43.67	18.99	17.14	17.72	34.8	31.59	31.37	0.39
	2	43.6	32.12	35.75	10.39	8.37	9.01	27.46	22.71	22.73	0.34
	3	**48.15**	**42.65**	**44.28**	**19.46**	**17.93**	**18.35**	**35.48**	**32.86**	**32.46**	**0.41**

The highest result is given in bold.

**Table 5:** Performance comparisons of the proposed system by using AraBERT preprocess and without using it.

Dataset	Preprocess	ROUGE-1			ROUGE-2			ROUGE-L			BLEU
		Precision	Recall	F1	Precision	Recall	F1	Precision	Recall	F1
AHS	With AraBERT	**54.95**	**50.48**	**51.49**	**13.1**	**12.01**	**12.27**	**37.84**	**35.19**	**34.37**	**0.41**
	Without AraBERT	52.44	49.5	49.79	12.23	11.5	11.6	36.12	34.66	33.22	0.39

AMN	With AraBERT	**48.15**	**42.65**	**44.28**	**19.46**	**17.93**	**18.35**	**35.48**	**32.86**	**32.46**	**0.41**
	Without AraBERT	45.9	38.3	40.7	17.63	15.53	16.18	33.19	29.21	29.15	0.36

The highest result is given in bold.

**Table 6:** Performance comparisons of the proposed system using several word embedding models.

Dataset	word2vec	ROUGE-1			ROUGE-2			ROUGE-L			BLEU
		Precision	Recall	F1	Precision	Recall	F1	Precision	Recall	F1	
AHS	Skip-gram	**54.95**	**50.48**	**51.49**	**13.1**	**12.01**	**12.27**	**37.84**	**35.19**	**34.37**	**0.41**
	CBOW	52.25	48.22	49.08	11.11	10.26	10.46	36.13	33.73	32.85	0.37

AMN	Skip-gram	**48.15**	**42.65**	**44.28**	**19.46**	**17.93**	**18.35**	**35.48**	**32.86**	**32.46**	**0.41**
	CBOW	43.78	38.83	40.11	17.05	15.21	15.92	32.28	29.7	29.03	0.38

**Table 7:** Comparison of research studies [13] and [9] with our proposed system.

Models	Year	ROUGE-1	Dataset
Dima Suleiman et al. [13]	2020	38.4	Author's dataset
Al-Maleh and Desouki [9]	2020	44.23	AHS
Our proposed system	2021	**51.49**	AHS

The highest result is given in bold.

**Table 8:** Two examples of generated summaries by the proposed system.

Example		
1	Article content	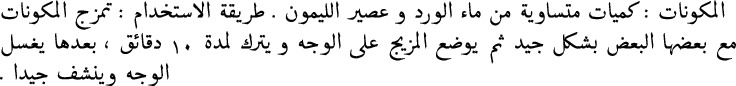
		Ingredients: equal amounts of rose water and lemon juice. How to use: mix the ingredients with each other well, then put the mixture on the face and leave for 10 minutes, then wash the face and dry it well.
	Reference summary	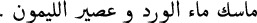
		Rose water and lemon juice mask.
	Generated summary	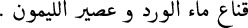
		Rose water and lemon juice mask.

2	Article content	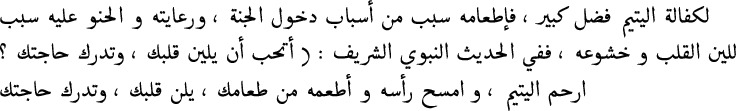
		Orphan Sponsoring has a great virtue; Feeding him is one of the reason to enter Paradise, Also caring of, and kindness to, an orphan cause the heart softness and reverence, in the honourable hadith of the Prophet: “IF you want to soften your heart and meet your needs, then have mercy on the orphan, wipe his head and feed him from your food, your heart will soften, and realize your need.
	Reference summary	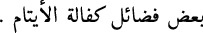
		Some of the virtues of sponsoring orphans.
	Generated summary	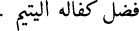
		The virtue of sponsoring an orphan.

3	Article content	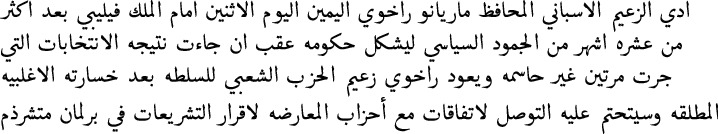
		This Monday, the Conservative Spanish leader, Mariano Rajoy, swore an oath by the King Felipe after more than ten months of political deadlock to form a government after the results of the two elections were inconclusive Rajoy, the leader of the People's Party, returns to power after losing the absolute majority. He will have to reach agreements with opposition parties to pass legislation in a fragmented parliament.
	Reference summary	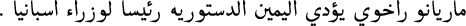
		Mariano Rajoy is sworn in as Prime Minister of Spain.
	Generated summary	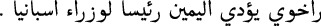
		Rajoy sworn in as Spain's prime minister.

## Data Availability

The data used to support the findings of this study were obtained from the Arabic Headline Summary (AHS) dataset (https://osf.io/btcnd/) and the Arabic Mogalad_Ndeef (AMN) dataset (https://drive.google.com/file/d/12Lqej0BcPelRQ81ewYrqkIl2xzfQald8/view).
